# Scoping Review of Available Culinary Nutrition Interventions for People with Neurological Conditions

**DOI:** 10.3390/nu16030462

**Published:** 2024-02-05

**Authors:** Chian Thong (Nicole) Chun, Lesley MacDonald-Wicks, Coralie English, Natasha A. Lannin, Amanda Patterson

**Affiliations:** 1 School of Health Sciences, Faculty of Health, Medicine and Wellbeing, University of Newcastle, Callaghan, NSW 2308, Australia; chianthong.chun@uon.edu.au (C.T.C.); lesley.wicks@newcastle.edu.au (L.M.-W.); coralie.english@newcastle.edu.au (C.E.); 2 Hunter Medical Research Institute Food and Nutrition Research Program, New Lambton Heights, NSW 2305, Australia; 3 Hunter Medical Research Institute Heart and Stroke Research Program, New Lambton Heights, NSW 2305, Australia; 4 Department of Neuroscience, Monash University, Melbourne, VIC 3004, Australia; natasha.lannin@monash.edu; 5 Allied Health, Alfred Health, Melbourne, VIC 3004, Australia

**Keywords:** stroke, rehabilitation, dietary interventions, dietary guidelines, nutrition intervention design, implementation, occupational therapy, stroke care

## Abstract

People with neurological conditions may face barriers to meal preparation. Culinary nutrition interventions aim to facilitate the building of knowledge and skills for meal preparation. This scoping review aims to map the available evidence for culinary nutrition interventions for people with neurological conditions and evaluate the quality of these interventions based on program design, delivery and evaluation. After a systematic search of online databases (MEDLINE, CINAHL, Embase, Scopus and Proquest) and reference lists, a total of ten publications describing nine interventions were included. Most interventions were designed for people with stroke and/or Transient Ischemic Attack (*n* = 3) and Multiple Sclerosis (*n* = 3); others were for traumatic brain injury (*n* = 1), mild dementia (*n* = 1) and Parkinson’s Disease (*n* = 1). Overall, the included culinary nutrition interventions had good program delivery (inclusion of motivational experiences, delivered by appropriate health providers) but needed improvements in program design (lack of consumer engagement and neurological symptom accommodations) and evaluation (lack of complete process, outcome and impact evaluations). In conclusion, the evidence base for culinary nutrition interventions for people with neurological conditions remains sparse. To bridge the gap between theory and practice, it is important to consider the following aspects in culinary nutrition intervention planning/improvement: (I) the involvement of consumers; (II) the accommodation/tailoring for post-condition effects; and (III) the coverage of all disease-specific culinary nutrition aspects.

## 1. Introduction

Living with a neurological condition (its post-condition effects, disease progression and drug–food interactions) can significantly impact an individual’s nutrition and nutritional state [1]. Neurological conditions are chronic conditions that affect the nervous system, including the brain and spinal cord, and the nerves that connect them [2]. It is an umbrella term for more than 600 diseases such as stroke, Multiple Sclerosis (MS), Parkinson’s Disease (PD), Alzheimer’s Disease, epilepsy and Traumatic Brain Injuries (TBI) [2].

Current scientific evidence suggests that dietary intake plays an important role in managing neurological conditions (e.g., preventing malnutrition and managing disease side effects). The European Society for Clinical Nutrition and Metabolism (ESPEN) clinical guidelines recommend people with neurological conditions engage with disease-specific dietary strategies tailored to their condition [1]. For instance, ESPEN recommends people with MS consume adequate omega-6 fatty acids to potentially decrease the number and severity of MS relapses (strong consensus with 100% agreement from the evidence base) [1]. People with PD are recommended to closely monitor their nutritional status (particularly vitamins D, B12 and folate) because these nutrient levels can be influenced by PD medications [1]. Recent research findings also aligned with ESPEN guidelines, suggesting that disease-specific dietary strategies are beneficial for optimal management of neurological conditions [3,4,5]. A recent review and a large-scale cohort study (60,000 participants) both confirmed that adherence to a Mediterranean diet was associated with improved cognitive performance and reduced risk of cognitive decline for people with Alzheimer’s and Parkinson’s Disease [3,5]. Furthermore, reducing dietary saturated fats may decrease the risk of dementia progression [3]. A recent systematic review (English et al.) also found that modifying certain dietary patterns is linked to reduced secondary stroke risk factors [4]. These include higher adherence to a Mediterranean-style diet (or at least increased intake of fruits, vegetables and fibres) and avoiding excessive salt intake [4].

Although research has suggested various dietary strategies to manage neurological conditions, people with these conditions struggle to implement these due to the lack of well-designed nutrition education programs that adequately accommodate for clinical symptoms and outcomes. This was reported in a recent scoping review by Russell et al. (2022), which found that there was limited evidence of nutrition education programs for people with neurological diseases [6]. Many published nutrition education programs also did not meet best-practice principles (i.e., not delivered by trained professionals, lack of appropriate evaluation processes) [6]. Eating well for neurological conditions means consuming nutritionally appropriate meals tailored to an individual’s condition. To eat well, people not only need nutrition knowledge, but also need to acquire the skills for performing various culinary nutrition tasks. Culinary nutrition tasks include planning meals, sourcing ingredients and cooking, compiling and storing meals. However, the effects of neurological conditions can make acquisition of these skills challenging. 

Culinary nutrition is the application of nutrition knowledge combined with hands-on cooking skills to create nutritious and fulfilling meals [7]. Culinary nutrition programs have so far been used for a limited number of varied population groups. For example, ‘Cooking for Vitality’ is a culinary nutrition intervention program that has been shown to significantly improve cancer-related fatigue (among cancer survivors) within two sessions of intervention [8]. There is also evidence that culinary nutrition intervention programs have been effective for people with diabetes [9], the general healthy population [10] and Indigenous communities living in Australia [11]. However, there is limited evidence for people with neurological conditions. 

Hence, this scoping review aimed to map the available culinary nutrition interventions for people with neurological conditions, and to evaluate the quality of these interventions based on program design, delivery and evaluation. 

## 2. Materials and Method

### 2.1. Methodological Framework

This scoping review was conducted using the methodological framework for scoping reviews proposed by Arksey and O’Malley (2005) and the Preferred Reporting Items for Systematic reviews and Meta-Analyses extension for Scoping Reviews (PRISMA-ScR) Checklist [12,13].

### 2.2. Eligibility Criteria

Studies were included if they:

1. Included participants with neurological conditions (nervous system diseases, stroke, brain injuries, Multiple Sclerosis, Parkinson’s Disease and Alzheimer’s Disease etc.);

2. Met our criteria for a ‘culinary nutrition intervention’.

To qualify as a ‘culinary nutrition intervention’, the main goal of the intervention needed to be to develop participants’ culinary nutrition literacy. Culinary nutrition literacy can be defined according to four areas (adopted from Vidgen et al., 2014): ‘Plan & manage’, ‘Select’, ‘Prepare’ and ‘Eat’ [14]. ‘Plan & manage’ includes the ability to prioritise money and time for food, to plan and ensure regular accessibility to food intake and to make feasible food decisions that balance food needs with available resources [14]. ‘Select’ is the ability to determine the advantages, disadvantages, ingredients, origin, storage, usage and quality of food products [14]. ‘Prepare’ is the ability to have sufficient skills in making meals from available resources (food products and culinary equipment) while applying basic safe food handling and hygiene principles [14]. ‘Eat’ is the ability to understand the impact of meals on personal well-being, to demonstrate self-awareness of the necessity of having a balanced meal intake, and to join and eat socially [14]. In addition, the culinary nutrition intervention needs to facilitate the development of cooking skills (cooking method and food preparation techniques) and/or food skills (meal planning, shopping, budgeting, resourcefulness and label reading) (adopted from Lavelle et al., 2017) [15]. Cooking skills are the physical or mechanical skills used to produce a meal (e.g., boiling water and peeling vegetables), as well as the conceptual and perceptual skills (e.g., understanding the transformation food undergoes when heat is applied, i.e., knowing that food is fully cooked from its colour, etc.) [15]. Food skills are the knowledge and skills to be able to prepare nutritionally and personally satisfying meals with the available resources [15].

We included all types of interventions (e.g., education, rehabilitation, nutrition therapy) unless they were not designed to support neurological condition survivors in a long-term home setting. For example, interventions designed for a hospital setting were excluded.

Additional exclusion criteria were as follows: all participants <18 years old; grey literature; review papers; and non-English-language articles. 

### 2.3. Information Sources and Search

Comprehensive literature searches for potentially relevant articles were conducted in the following five online databases: MEDLINE, CINAHL, Embase, Scopus and Proquest during May 2023. Search strategies from each online database are included in Supplementary Table S1. Researchers developed the initial search strategy in MEDLINE (Supplementary Table S1). Similar search strategies were used to search the other identified databases. The final search results were exported into EndNote 20.2.1 referencing software [16]. After removing duplicates, the results were uploaded into the online systematic review management system Covidence [17] for article screening purposes. Reviewers also hand-searched the reference lists of the final included studies for additional publications (August 2023).

### 2.4. Selection of Sources of Evidence

After the removal of duplicates from EndNote 20.2.1 [16] and Covidence [17], two authors (CTC and AP) double screened the first 20 records independently, discussed the results and resolved conflicts with a third author (LMW). Following this process, the inclusion/exclusion criteria were clarified and revised. This was to ensure consistency in the following screening process. Three authors single screened (CTC, AP or CE) the remaining records by their title and abstract. Potential full-text articles were double screened by two authors (CTC and AP), with conflicts resolved with LMW. Articles that met all inclusion criteria were included in this review. 

### 2.5. Data-Charting Process and Data Items

A standardised data-charting form (a customised spreadsheet) was designed by CTC to chart data extracted from eligible publications (copy available from senior author on request). The included variables in the data-charting form were study characteristics (authors, year, country of study, journal, study design), study population (type of neurological condition, participants number and characteristics), characteristics of culinary nutrition intervention (name, aim of intervention, type of intervention, setting, group size, caregivers involvement, delivery personnel, duration, inclusion of behavioural change techniques, tailoring of the intervention), research outcome (evaluation tools, comparator, intervention outcome) and targeted culinary nutrition components (culinary nutrition content within the intervention, embedded knowledge provision/skills training, embedded food skills/cooking skills content, targeted culinary nutrition areas). These variables were included to map the culinary nutrition interventions and to analyse the program design (i.e., tailoring to disease-specific content, evidence-based, inclusion of culinary nutrition content), program delivery (i.e., inclusion of motivational experiences, educator characteristics) and program evaluation (i.e., inclusion of process, outcome and impact evaluations related to culinary nutrition knowledge and skills).

The culinary nutrition content of each intervention was analysed based on the culinary nutrition literacy components (Vidgen et al., 2014) [14], culinary nutrition skills (Lavelle et al., 2017) [15] and behavioural change motivational experiences (Fredericks et al., 2020). Fredericks et al., 2020, identified ten motivational experiences that can motivate sustainable behaviour change in culinary nutrition interventions [18]. They determined that if these ten motivational experiences (Challenge, Celebration, Collaboration, Home Environment, Palate Development, Peer Support, Recipe Concept, Skill Building, Skill Reinforcement and Success) were experienced by participants during the intervention, it would effectively motivate them to develop the intended culinary nutrition-related skills [18]. The definition of each motivational experience is detailed in Table 1. 

### 2.6. Synthesis of Results

Results were synthesised narratively and are presented in Table 2, Table 3 and Table 4, and summarised in Table 5. We assessed the quality of the culinary nutrition content of the interventions based on the following criteria:

(1) Program design (i.e., tailoring to disease-specific content, evidence-based, inclusion of culinary nutrition content).

(2) Program delivery (i.e., inclusion of motivational experiences, educator characteristics).

(3) Program evaluation (i.e., inclusion of process, outcome and impact evaluations related to culinary nutrition knowledge and skills).

## 3. Results

### 3.1. Included Studies

**Figure 1:**
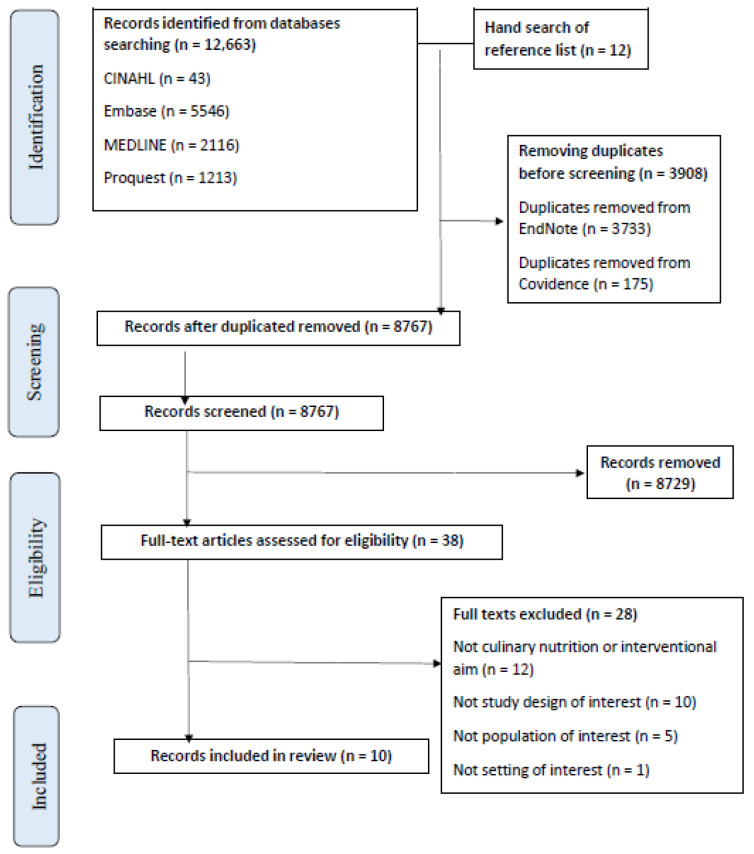
The Preferred Reporting Items for Systematic reviews and Meta-Analyses (PRISMA) flowchart.

A total of 12,675 articles were retrieved using the search strategy (Supplementary Table S1). Once duplicates were removed, 8675 articles were single screened by title and abstract in Covidence [17] (CTC, AP, CE). The eligible 38 full-text articles were double screened (CTC, AP). We excluded 28 full-text articles due to them being: not an aim of interest (*n* = 12), not a study design of interest (*n* = 10), not a population of interest (*n* = 5) and not a setting of interest (*n* = 1). A total of ten eligible studies had data extracted for this scoping review [19,20,21,22,23,24,25,26,27,28], which included a total of nine culinary nutrition interventions (as one intervention was published across two articles [20,21]). The Preferred Reporting Items for Systematic reviews and Meta-Analyses (PRISMA) flowchart (Figure 1) reports the flow of records into the review (Figure 1).

### 3.2. Study Characteristics (Table 2)

Most studies (*n* = 3, 33%) included people with stroke and/or Transient Ischemic Attack (TIA) [19,20,21,22], including one study specifically in stroke survivors living with dysphagia [19], or MS (*n* = 3, 33%) [23,24,25]. The remaining three studies included participants diagnosed with traumatic brain injury (TBI) [26], mild dementia [27] and Parkinson’s Disease (PD) [28]. 

Most studies were conducted in the United States (*n* = 5, 56%) [20,21,22,23,26,28], followed by Asian countries (*n* = 2, 22%) [19,27], with one study each from the United Kingdom [24] and Australia [25]. Most articles were published in the last five to ten years (*n* = 6, 67%) [19,20,21,23,25,27,28], while those remaining were published more than ten years ago (*n* = 3, 33%) [22,24,26]. In terms of study designs, all included studies (*n* = 9, 100%) can be classified as pilot study designs, thus having relatively small sample sizes (4 to 100 participants; mean 33) [19,20,21,22,23,24,25,26,27,28]. The majority of participants were 40 years old and above (*n* = 8, 89%) [19,20,21,23,24,25,27,28] except for the study involving people with TBI [26]. Almost half of the studies recruited more than 50% male participants (*n* = 4, 45%) [19,20,21,26,29], while two studies recruited more than 50% females (*n* = 2, 22%) [22,24] and one study recruited solely female participants with Multiple Sclerosis [23]. Almost half (*n* = 4, 45%) of the interventions reported the involvement of caregivers [19,25,27,28], while one intervention involved health professionals (neurologist, nurse, dietitian, clinical psychologist, researcher) as stakeholders during the program design phase [25]. Two interventions (reported in three studies) recruited people from culturally and linguistically diverse backgrounds (Spanish-speaking Americans [20,21] and African Americans [22]).

**Table 2:** Study characteristics of included studies.

No.	Authors, Year	Study Characteristics		Neurological Condition	Participants’ Characteristics		
		Journal, Country	Study Design		Total Number of Participants	Age in Years (Mean ± SD or Range)	%Female; Other Characteristics
1	Lin, Shu-Chi et al., 2021 [19]	Medicine Journal, Taiwan	Pilot, RCT, single-blinded	Stroke (dysphagia)	*n* = 22	78 ± 8	36; caregivers involved
2	Amytis Towfighi et al., 2020 [20]	Journal of Stroke and Cerebrovascular Diseases, US	Pilot, RCT	Stroke and TIA	*n* = 100	58 ± 9	38; 60% spanish-speaking
3	Valerie A. Hill et al., 2017 [21]	Journal of Stroke and Cerebrovascular Diseases, US					
4	James H. Rimmer et al., 2020 [22]	American Journal of Preventive Medicine, US	Pilot, pre–post	Stroke	*n* = 62	53 ± 8	75; predominantly urban African-American population, 37% had hemiplegia and 74% used a cane to ambulate
5	Ilana Katz Sanda et al., 2019 [23]	Multiple Sclerosis and Related Disorders, US	Pilot, RCT	Multiple Sclerosis	*n* = 36	43 (32–51)	100
6	Mary J. Doidge, 1993 [24]	Journal of Human Nutrition and Dietetics, UK	Pilot, pre–post	Multiple Sclerosis	*n* = 48	47 ± 10	60
7	Rebecca D. Russell et al., 2023 [25]	Disability and Rehabilitation, Australia	Pilot, mixed-method, co-design	Multiple Sclerosis	Phase 1: *n* = 114; Phase 2: *n* = 16; Phase 3: *n* = 8	Phase 1: 52 ± 12; Phase 2: NR; Phase 3: 39 ± 11	Phase 1: 20, Phase 2: NR; stakeholders included, Phase 3: 22; caregivers included
8	M. McGraw-Hunter et al., 2009 [26]	Brain Injury, US	Pilot, pre–post	Traumatic brain injury (TBI)	*n* = 4	27	25 (All Caucasian)
9	Min-Soo Cho et al., 2019 [27]	Journal of Exercise Rehabilitation, Korea	Pilot, pre–post	Mild dementia	*n* = 23	84 ± 5	NR; caregivers involved
10	Priscilla Brenes et al., 2021 [28]	Thesis Dissertation, US	Pilot, mixed-method, pre–post	Parkinson’s Disease	*n* = 27	67	NR; caregivers involved

Abbreviation: NR: Not Reported; RCT: Randomised Controlled Trial; MS: Multiple Sclerosis; TIA: Transient Ischemic Attack.

### 3.3. Characteristics of Included Interventions (Table 3)

The included culinary nutrition interventions were delivered in several formats: food preparation programs (stroke) [19], nutrition education embedded in a lifestyle modification intervention (stroke, mild dementia) [20,21,22,27], nutrition or dietary education (MS) [23,24], online nutrition learning modules (MS, PD) [25,28] and video self-modelling (TBI) [26]. Most interventions were delivered in small groups (three to eleven participants per group) [19,20,21,23,24], with a few studies delivered via self-paced learning modules [25,26]. Almost all interventions were delivered by relevant health professionals, including dietitians [19,22,23,24,25], occupational therapists [20,21], physiotherapists [24] and researchers [25,26,28]. The duration of interventions varied from six weeks to six months; most interventions were scheduled by an organiser [19,20,21,22,23,24,26,27], while some were designed to be self-paced [25,28] or a combination of both [26]. All included studies tailored their interventions specifically to the participants’ neurological condition [19,20,21,22,23,24,25,26,27,28], living situation (socioeconomic situation) [22] or learning interest (personal goals) [20,21].

The identification of Fredericks et al.’s (2020) motivational experiences (Challenge, Celebration, Collaboration, Home Environment, Palate Development, Peer Support, Recipe Concept, Skill Building, Skill Reinforcement and Success [18]) was analysed based on the reported intervention content. All interventions had included four or more motivational experiences to motivate participants’ behavioural change [19,20,21,22,23,24,25,26,27,28], while only one stroke program (reported in two studies) [20,21] utilised all ten types of motivational experiences in the intervention. All interventions utilised the experiences of ‘Challenge’ and ‘Skill Building’ (*n* = 9, 100%) [19,20,21,22,23,24,25,26,27,28] and 89% (*n* = 8) of studies included the experience of ‘Success’ [19,20,21,23,24,25,26,28]. There were 67% (*n* = 6) of the included studies that used experiences of ‘Home Environment’ [20,21,22,23,24,26,28], ‘Palate Development’ [19,20,21,22,23,24,25] and ‘Recipe Concept’ [19,22,23,24,25,27,28]; 56% (*n* = 5) utilised the ‘Skill Reinforcement’ [20,21,22,23,25,26] experience. Only two interventions (reported in three studies) used ‘Collaboration’ [20,21,25] and ‘Peer Support’ [20,21,25], and only one intervention (reported in two studies) reported using the ‘Celebration’ experience in their program planning [20,21].

**Table 3:** Characteristics of included interventions.

No.	Authors, Year	Characteristics of Intervention					
		Format	Group Size	Educator(s)	Duration	Tailoring	Motivational Experiences
1	Lin, Shu-Chi et al., 2021 [19]	Food Preparation Program	3 to 4	Dietitian	6 weeks (frequency NR)	Food and drink texture modification; disease-specific content	Challenge, Palate Development, Recipe Concept, Skill Building, Success
2	Valerie A. Hill et al., 2017 [21]	Lifestyle Education Program (Embedded Nutrition Education)	3 to 8	Occupational therapist	6 weeks (weekly 2 h session)	Personal learning goals; disease-specific content	Challenge, Celebration, Collaboration, Home Environment, Palate Development, Peer Support, Recipe Concept, Skill Building, Skill Reinforcement, Success
3	Amytis Towfighi et al., 2020 [20]						
4	James H. Rimmer et al., 2000 [22]	Health Promotion Intervention (Embedded Nutrition Education)	NR	Dietitian (nutrition classes); other relevant allied health professional for other classes	12 weeks (3 days a week)	Socioeconomic situation; disease-specific content	Challenge, Home Environment, Palate Development, Recipe Concept, Skill Building, Skill Reinforcement, Success
5	Ilana Katz Sanda et al., 2019 [23]	Dietary Education	5	Dietitian	6 months (frequency NR)	Disease-specific content	Challenge, Palate Development, Recipe Concept, Skill Building, Skill Reinforcement, Success
6	Mary J. Doidge, 1993 [24]	Nutrition Education Programme	8 to 11	Dietitian (nutrition session); physiotherapist (exercise session)	>8 weeks (8 weekly 90 min sessions)	Co-designed; disease-specific content	Challenge, Palate Development, Recipe Concept, Skill Building, Success
7	Rebecca D. Russell et al., 2023 [25]	Online Nutrition Education Modules	N/A; self-learning	People with MS and health professionals (dietitian, professor, etc.)	1 year (self-paced)	Co-designed; disease-specific content	Challenge, Collaboration, Palate Development, Peer Support, Recipe Concept, Skill Building, Skill Reinforcement, Success
8	M. McGraw-Hunter et al., 2009 [26]	Video Self-Modelling	N/A; self-learning	Researcher	4 weeks (4 training sessions)	Providing verbal prompts individualised to each participant	Challenge, Home Environment, Skill Building, Skill Reinforcement, Success
9	Min-Soo Cho et al., 2019 [27]	Exercise and nutrition education program	NR	NR	16 weeks (16 nutrition education sessions, 20 min each)	Ongoing check-in with health and nutrition issues; disease-specific content	Challenge, Recipe Concept, Skill Building
10	Priscilla Brenes et al., 2021 [28]	Online Nutrition Education Modules	NR	Instructor	8 weeks (6 modules)	Disease-specific content	Challenge, Recipe Concept, Skill Building, Success

Abbreviation: NR: Not Reported; N/A: Not Applicable; RCT: Randomised Controlled Trial; MS: Multiple Sclerosis; TIA: Transient Ischemic Attack; QoL: Quality of Life; SMART: Specific, Measurable, Achievable, Relevant, and Time-Bound.

### 3.4. Culinary Nutrition Components and Intervention Outcome (Table 4)

The details of the culinary nutrition content from each intervention can be found in Table 4. All included interventions were based on disease-specific culinary nutrition content [19,20,21,22,23,24,25,26,27,28] tailored to participants’ neurological condition. Most culinary nutrition interventions [19,20,21,22,23,24,25] provided both knowledge and skills training, two interventions focused on only knowledge provision for food skills [27,28] and one intervention focused on cooking skills training [26]. The majority (*n* = 7, 78%) of the interventions were designed to intervene for all aspects of Vidgen et al.’s (2014) culinary nutrition literacy areas (‘Plan & manage, Select, Prepare, Eat’) [19,20,21,22,23,24,25,28], while the remaining interventions focused on one or two culinary literacy areas (‘Select’ and ‘Eat’ [27] or ‘Prepare’ [26]).

In terms of program evaluation, all included articles reported their evaluation method, including both quantitative (*n* = 8, 89%) [19,20,21,22,23,24,26,27,28] and/or qualitative (*n* = 7, 78%) [19,20,21,22,23,24,25,28] measures. The quantitative measures evaluated participants’ dietary intakes [19,20,21,23,24,27,28], culinary nutrition skills or knowledge [26,28], biomedical measures [20,21,22] and/or the management of neurological side effects [19,23,28]. For dietary intakes, several methods were used: food frequency questionnaires [23], diet history/recall/record [20,21,23,24,28] and/or validated malnutrition detection questionnaires (e.g., Mini Nutritional Assessment) [19,27,28], or Mediterranean diet scores [23]. To evaluate the management of neurological side effects, the included studies used disease-specific measures such as dysphagia self-detection evaluations [19], MS-related fatigue and disability status evaluation [23] and PD-related bowel health evaluation [28]. The qualitative measures explored participants’ attitudes towards the program design. This included the evaluation of program adherence [20,21,23], qualitative discussions of the intervention sessions [20,21,24,25,28], self-reported health-related quality of life [19,22,28] and/or attitude changes towards meal preparation [24].

All studies produced positive outcomes, including the successful improvement in participants’ dietary quality and/or general well-being [19,20,21,22,23,24,26,27,28], Quality of Life (QoL) [19,22,28] or better management of side effects [22,23,28] after completing the interventions. The outcomes were either compared with a usual care control group [19,20,21,22,23] or based on pre- and post-data from the intervention group [24,25,26,27,28]. One intervention reported non-significant results for dietary and biomedical outcomes, but the focus groups determined the intervention as feasible and efficacious [20,21].

**Table 4:** The culinary nutrition components and intervention outcome from included articles.

No.	Authors, Year	Targeted Culinary Nutrition Components				Outcome		
		Culinary Nutrition Content	Knowledge Provision/Skills Training	Cooking Skills/Food Skills	Culinary Nutrition Literacy	Evaluation Tool(s)	Comparator	Intervention Outcome
1	Lin, Shu-Chi et al., 2021 [19]	Oral motor exercises, food texture and thickener recognition, hands-on food preparation, nutrition education. Choosing healthy food, maintaining a balanced diet, preparing ingredients, natural thickeners, energy-boosting foods, techniques of reshaping food, soft food recipes, a balanced and nutritious texture-modified diet.	Both	Both	Plan & manage, Select, Prepare, Eat	Dysphagia self-detection tool (EAT-10), Dietary Well-Being Questionnaire (brief version of the WHO QoL, Swallowing QoL Questionnaire and MNA) Health-related QoL Scale (WHOQOL-BREF)	Usual Care	Positive effects on patients’ self-perceived diet quality and well-being/QoL. Potential improvements in health-related QoL; QoL associated with the process of swallowing, and nutritional status.
2	Amytis Towfighi et al., 2020 [20]	Nutrition Education (‘Eating Healthy—introducing Mediterranean and DASH diet’, ‘Avoid Dietary Pitfalls’) Implementation practice (e.g., making green smoothies, preparing healthy salads, taking field trips to a local grocery store and restaurant with a small budget)	Both	Both	Plan & manage, Select, Prepare, Eat	Related body measures, dietary intake, program adherence, focus groups	Usual Care	Intervention shown to be feasible and efficacious. Insignificant outcome in dietary and biomedical measures.
3	Valerie A. Hill et al., 2017 [21]							
4	James H. Rimmer et al., 2000 [22]	The classes included ‘hands-on’ cooking instruction that focused on low-fat, low-cholesterol food items. Participants were taught how to cook healthy meals during the first two classes of the week and then cooked their own healthy meal during the third class. Group discussion in ‘Health Behaviour’ class.	Both	Both	Plan & manage, Select, Prepare, Eat	Biomedical, fitness, nutritional and psychosocial measures. Nutrition-related measures: dietary fat intake, LSQ22, SCL-90R.	Control Group	Treatment group reduced total cholesterol, weight, social isolation; increased cardiovascular fitness, strength, flexibility, life satisfaction and ability to manage self-care needs.
5	Ilana Katz Sanda et al., 2019 [23]	Nutrition education regarding healthy Mediterranean-style eating pattern for Americans (tips for grocery shopping, sample menu plan, reading food labels, eating in restaurants and travel) with access to registered dietitian’s guidance (meetings or emails).	Both	Both	Plan & manage, Select, Prepare, Eat	Various, including: dietary measure, e.g., Food Frequency Questionnaire, 3 dietary recalls, adherence to US-style Mediterranean diet score. Symptom evaluation, e.g., Neurological Fatigue Index-MS score, MS Impact Scale, Expanded Disability Status Scale. Program self-adherence	Non-Interventional Group	Excellent program self-adherence. The intervention group showed significant decline in fatigue and MS disease impact and disability status.
6	Mary J. Doidge, 1993 [24]	Topics in the program are Introductory Diet Analysis, Fat, Healthy Eating, Preparing Food at Home, Choosing Food Sensibly, Vitamins and Minerals, Lifestyles, Recipe Tasting.	Both	Both	Plan & manage, Select, Prepare, Eat	Seven-day weighed food and drink record. Participant’s dietary attitude assessment, subjective questionnaires (participants and dietitians)	No	(1) Dietary analysis: significant positive improvements in nutrient intakes. (2) Attitude change towards meal preparation: small increase because the majority of participants already had positive attitudes prior to the program. (3) Dietitians’ and participants’ subjective evaluation: All the dietitians felt the programme had gone well and all had enjoyed it themselves. Session aims and objectives had generally been met. Suggestions were made on several program topics.
7	Rebecca D. Russell et al., 2023 [25]	Topics in the intervention are Diet, MS Progression, MS Symptoms (Managing Fatigue in kitchen), Healthy Eating for MS, Assessing evidence, Putting into practice—meal planning and managing MS, Inflammation, Gut health, Depression, Future dietary research. Diet content, video, discussion board, workbook activities.	Both	Both	Plan & manage, Select, Prepare, Eat	Qualitative methods (survey, focus group, interviews)	No	Identified recommendations to the intervention; developed a full program prototype for feasibility study.
8	M. McGraw-Hunter et al., 2009 [26]	Participants received instruction in cooking stovetop meals (i.e., a boxed rice meal, stovetop noodles) at own home. They watched videotapes of themselves cooking and practiced that skill while receiving prompts and feedback.	Skill Training	Cooking Skills	Prepare	Ability to complete a 25-step recipe (percentage of completion)	No	Three of the four individuals achieved criterion performance (stovetop food preparation) within four training sessions; substantially maintained their skills 2- and 4-weeks following training and generalised their skills to a novel food item.
9	Min-Soo Cho et al., 2019 [27]	The main contents of nutrition education were divided into four fields: the concept of health, proper eating habits, nutrition and nutrients, and the problems of hypernutrition and nutrient deficiency.	Knowledge Provision	Food Skills	Select, Eat	MNA	No	Significant increase in MNA score (reduced risk of malnutrition)
10	Priscilla Brenes et al., 2021 [28]	General nutrition knowledge, Label reading, Parkinson’s Disease–diet relationship and tips.	Knowledge Provision	Food Skills	Plan & manage, Select, Prepare, Eat	Bowel health (BHQ), diet history (DHQ3), MNA, Nutrition Knowledge and Program Evaluation, Disease QoL (PDQ-39, UPDRS, CHAMPS, TSRQ)	No	Participant’s total consumption of macro- and micronutrients increased. A total of 50% of participants improved QoL scores. Participants more aware of healthy eating, gut health, hydration, food–medication interaction and constipation.

Abbreviation: NR: Not Reported; N/A: Not Applicable; WHO: World Health Organization; MNA: Mini Nutritional Assessment; LSQ-22: Life Satisfaction Questionnaire; SCL90R: Symptom Check List-90 Revised; RCT: Randomised Controlled Trial; MS: Multiple Sclerosis; TIA: Transient Ischemic Attack; QoL: Quality of Life; DASH: Dietary Approaches to Stop Hypertension.

### 3.5. Summary of Program Design, Delivery and Evaluation for Included Interventions (Table 5)

Table 5 was synthesised to provide a summary of results (from Table 2, Table 3 and Table 4) based on the aspects of program design, delivery and evaluation. Program design was assessed by the inclusion of disease-specific content, accommodation of neurological side effects, appropriate culinary nutrition content (i.e., skill training, knowledge provision, food and cooking skills training, aimed to improve culinary nutrition literacy), attendance flexibility and co-design process during intervention development. Program delivery was assessed by the utilisation of appropriate educator and motivational experiences during intervention delivery. Program evaluation was assessed by the completion of the process, impact and outcome evaluation after each intervention. Based on the 11 aspects identified from the above, all included interventions showed seven to nine positive aspects in their intervention [19,20,21,22,23,24,25,26,27,28]. For program design, all included interventions demonstrated the inclusion of appropriate content (disease-specific content, appropriate activities aimed to improve participants’ culinary nutrition knowledge or skills) [19,20,21,22,23,24,25,26,27,28]. Only two interventions accommodated neurological side effects [19,23]; two interventions offered flexibility in attendance [25,28]; and only one intervention was co-designed with consumers [25]. For program delivery, most interventions (*n* = 7, 78%) were conducted by appropriate educators such as dietitians and occupational therapists [19,20,21,22,23,24,25]. All included interventions utilised three or more Fredericks et al., 2020, motivational experiences [19,20,21,22,23,24,25,26,27,28], but only one intervention utilised all types of motivational experiences [20,21]. For program evaluation, all included interventions evaluated their program, but only three completed the process, impact and outcome evaluation [23,24,28].

**Table 5:** Summary of program design, delivery and evaluation for included interventions.

No.	Culinary Nutrition Intervention	Program Design							Program Delivery		Program Evaluation (Process, Impact, Outcome)	Positive Outcome
		Disease-Specific Content	Side Effects Accommodation	Skills/Knowledge	Food/Cooking Skills	Culinary Nutrition Literacy	Attendance Flexibility	Co-Design	Appropriate Educator	Motivational Experiences		
1	Lin, Shu-Chi et al., 2021 [19]	√	√ (Dysphagia)	√ (Both)	√ (Both)	√ (All)	X	X	√ (Dietitian)	√ (Lacking Celebration, Collaboration, Peer Support, Skill Reinforcement)	√ (Impact, Outcome)	√ (Dietary quality, QoL, side effect management, nutritional status)
2	Valerie A. Hill et al., 2017 [20]	√	X	√ (Both)	√ (Both)	√ (All)	X	X	√ (Occupational therapist)	√ (All)	√ (Process, Impact)	√ (Feasibility and efficacy of program)
3	Amytis Towfighi et al., 2020 [21]											
4	James H. Rimmer et al., 2000 [22]	√	X	√ (Both)	√ (Both)	√ (All)	X	X	√ (Dietitian)	√ (Lacking Celebration, Collaboration)	√ (Impact, Outcome)	√ (Biomedical measures related to cardiovascular health, life satisfaction, self-management ability)
5	Ilana Katz Sanda et al., 2019 [23]	√	√ (Fatigue)	√ (Both)	√ (Both)	√ (All)	X	X	√ (Dietitian)	√ (Lacking Celebration, Collaboration, Peer Support)	√ (All)	√ (Program self-adherence, fatigue, MS disease impact and disability status)
6	Mary J. Doidge, 1993 [24]	√	X	√ (Both)	√ (Both)	√ (All)	X	X	√ (Dietitian)	√ (Lacking Celebration, Collaboration, Peer Support, Skill Reinforcement)	√ (All)	√ (Nutrient intakes, attitudes towards diet, program design)
7	Rebecca D. Russell, 2023 [25]	√	X	√ (Both)	√ (Both)	√ (All)	√	√	√ (Dietitian, people with MS)	√ (Lacking Celebration, Home Environment)	√ (Process)	NR (Developed a full program prototype)
8	M. McGraw-Hunter et al., 2009 [26]	√	X	√ (Skills training)	√ (Cooking)	√ (Prepare)	X	X	NR (Researcher)	√ (Lacking Celebration, Collaboration, Palate Development, Peer Support)	√ (Impact)	√ (Sustainable stovetop food preparation skills)
9	Min-Soo Cho et al., 2019 [27]	√	X	√ (Knowledge provision)	√ (Food)	√ (Select, Eat)	X	X	NR	√ (Lacking Celebration, Collaboration, Home Environment, Palate Development, Peer Support, Skill Reinforcement, Success)	√ (Impact)	√ (Nutrition condition)
10	Priscilla Brenes, 2021 [28]	√	√ (Gut health, inflammation)	√ (Knowledge Provision)	√ (Food)	√ (All)	√	X	NR (Instructor)	√ (Lacking Celebration, Collaboration, Palate Development, Peer Support, Skill Reinforcement)	√ (All)	√ (Diet quality, QoL, awareness of food and disease relationship)

Abbreviation: √: A tick if the intervention met the criteria; X: A cross if the intervention did not meet the criteria; NR: Not Reported; QoL: Quality of Life.

## 4. Discussion

While developing culinary nutrition skills and knowledge are important following diagnosis with or rehabilitation for a neurological condition, few interventions were located for this scoping review. Our findings are similar to a recent scoping review on nutrition education programs, which also concluded that culinary nutrition interventions are lacking [6]. There were only ten publications, divided among stroke, MS, mild dementia, PD and TBI. Similar to Russell et al.’s (2022) scoping review, we found no culinary nutrition interventions for people with epilepsy, Huntington’s Disease or motor neuron disease, where all these patient groups could benefit from tailored culinary nutrition programs [30,31,32,33].

This scoping review found ten articles (reporting nine interventions) that had implemented culinary nutrition interventions for adults with neurological conditions. Overall, it was found that the delivery of these programs could be considered good but that improvements were needed in the design and evaluation of the interventions.

In terms of program delivery, it was positive to observe that most culinary nutrition interventions were delivered by appropriate health professionals (dietitians and occupational therapists), which was different from the findings of the Russell et al. 2022 review [6] on nutrition education for neurological diseases. The incorporation of motivational experiences that facilitate behavioural change was also evident in all interventions. These might have contributed to the successes (i.e., improved dietary quality and/or general well-being, improved QoL and better side effect management) seen for the included interventions. Although most programs were delivered in groups, there was a lack of guided peer support or collaborative activities in the included interventions. These activities were found to be encouraging for participants during the rehabilitation process for people with TBI, stroke, PD or MS [34]. Future programs should, therefore, consider the addition of these experiences for better outcomes. The optimal duration and frequency for a culinary nutrition intervention was inconclusive as the included interventions were either embedded within other health programs, were self-paced or did not report these data.

In terms of program design, all included culinary nutrition interventions had tailored their programs to align with evidence-based recommendations from recent clinical guidelines and research [1,3,5]. However, only a few interventions [19,23,28] addressed the accommodation of neurological side effects (dysphagia, fatigue, gut health, inflammation), which may be a barrier to good nutrition [35]. The included culinary nutrition interventions were generally not personalised enough to be adequately tailored for the participants’ individualised recovery journey. Most interventions were delivered via scheduled sessions, resulting in limited flexibility for participants to attend and engage regularly, especially considering the additional barriers from neurological side effects. People from culturally and linguistically diverse backgrounds are at increased risk of poor nutrition due to additional multifactorial barriers (socioeconomic, healthcare systems and providers) in accessing healthcare and healthier food choices [36,37,38]. However, there were only two stroke programs [20,21,22] that included culturally and linguistically diverse (Spanish-speaking, African American) participants in the United States, showing that more work needs to be carried out in these areas. The importance of consumer engagement and co-design in healthcare interventions has increasingly been recognised in recent years [29]. Consumer engagement and co-design have shown potential in aligning health services with consumer needs, and improving engagement and uptake with healthcare [29]. Consumers include the people who receive the care, people with lived experience, people who provide care or decision makers [29]. Within the current evidence base, there was only one MS online learning intervention [25] that utilised a co-design approach, meaning 90% of the culinary nutrition interventions were not co-designed with any consumer partners, and were solely planned by researchers.

Research shows that completing all stages of evaluation (process, impact and outcome) can result in a more engaging health-related behavioural change intervention [39]. Most studies only completed either one or two stages of evaluation, with appropriate culinary nutrition-related measures. For instance, quality of intervention and participant engagement were measured in process evaluation; dietary and biomedical measures were utilised in impact evaluation; and quality of life and meal preparation attitudes were evaluated in outcome evaluation. Among all the included studies, only one study considered the sustainability of behaviour change [26]. As culinary nutrition skills can be improved by sustained behavioural change, future studies should consider evaluating their outcome sustainability and provide future directions in this area.

Among the limited publications, all the included culinary nutrition interventions were delivered by appropriate health professionals using evidence-based content. More work needs to be carried out in peer support activity planning, the accommodation of side effects and cultural barriers, consumer engagement and evaluating outcome sustainability.

There were a few limitations in this scoping review, with the small number of eligible studies being the main one. Due to the small number of eligible studies, this scoping review included diverse study designs and methodologies, leading to heterogeneity in the data. Due to the limited data, the critical appraisal of study quality was omitted, which may limit the depth of our findings. Additionally, all included interventions had short study periods (less than a year) and small sample sizes (*n* = 4 to 100), which are barriers to analysing the outcomes of culinary nutrition interventions. Publication bias is another possible limitation given the small number of studies found and the mostly positive reported outcomes. As evidenced by the fact that included studies were mostly recent publications, research in the area of culinary nutrition programs is growing rapidly. Hence, this scoping review may need to be repeated in the near future and should include a search of registered trials to capture the full breadth and scope of the research activity. All the above limitations may limit the generalisability of our findings.

Despite these limitations, this was a thorough scoping review and mapped all the available culinary nutrition interventions for multiple types of neurological conditions at this time. Substantial work was undertaken to evaluate each of the interventions in relation to the program design, delivery and evaluation.

## 5. Conclusions

Culinary nutrition interventions for neurological conditions are a complicated area of development due to the complexity, variety and individuality of diagnoses and disease progression. The availability of evidence-based culinary nutrition programs is lacking in many neurological conditions with only limited numbers in stroke, MS, PD, dementia and TBI. It is promising, however, to observe the increased number of studies in recent years, with more focused on living well with the neurological conditions. To bridge the gap between theory and practice, it is important to consider these aspects in culinary nutrition intervention planning/improvement: (I) the involvement of consumers; (II) the accommodation/tailoring of post-condition side effects; (III) and the coverage of all disease-specific culinary nutrition aspects (culinary nutrition literacy components (Vidgen et al., 2014), culinary nutrition skills (Lavelle et al., 2017) and behavioural change motivational experiences (Fredericks et al., 2020)). More research is needed in the areas of stroke, MS, TBI, mild dementia and PD. In addition, there are many neurological conditions without any current evidence base, including but not limited to Epilepsy, Alzheimer’s Disease and Huntington’s Disease.

## Figures and Tables

**Table 1:** Motivational experiences for culinary nutrition education that motivate behaviour change (adopted from Fredericks et al., 2020) [18].

Motivational Experiences	Definition
Challenge	Encourage participants to move out of their ‘comfort zone’ by exploring new foods/flavours/skills via their taste, smell, texture and sound
Celebration	Create a fun, enjoyable and special atmosphere; create deliciousness from nutritious meals to encourage participants to enjoy the taste and try new things
Collaboration	Generate a positive group dynamic, values group accomplishments, sharing positive feelings about food with peers; create a feeling of being a part of something bigger
Home Environment	Actively addressing home dynamics, facilities and access to nutritious meals; create solutions and strategies tailored to own home environment
Palate Development	Explore, investigate and taste a wide range of flavours from fresh ingredients, spices and condiments; build anticipation and excitement in new combinations
Peer Support	Create a supportive environment among peers with similar experiences; normalise and accept new behaviours
Recipe Concept	Move beyond recipe-driven cooking, encourage participants to utilise recipe concepts; swap ingredients according to availability
Skill Building	Build culinary nutrition-related skills, motivate participants to share their learnings with others
Skill Reinforcement	Reinforce learnt skills via repetitive prompt or performance assessments over multiple sessions
Success	Create activities with small steps, increase participants’ confidence and competency, develop a sense of accomplishment

## Supplementary Materials

The following supporting information can be downloaded at: https://www.mdpi.com/article/10.3390/nu16030462/s1, Table S1: Search Strategies.
