# Supplementary material for: Scoping Review of Available Culinary Nutrition Interventions for People with Neurological Conditions
Source: Nutrients. 2024 Feb 5;16(3):462. doi: 10.3390/nu16030462 (PMC10857153; doi:10.3390/nu16030462)
Supplement: Supplementary file 1 [file nutrients-16-00462-s001.zip › nutrients-2788968-supplementary.pdf]

Supplementary Material

Supplementary Table S1: Search Strategies

No.	Search Terms (MEDLINE)	Results (n = 2,116)
1	Nervous System Disease/ or neurological condition.mp.	50,112
2	stroke.mp. or Stroke/	366,306
3	Brain Injuries/ or brain injur*.mp.	110,724
4	multiple sclerosis.mp. or Multiple Sclerosis/	98,311
5	Parkinson Disease.mp. or Parkinson Disease/	90,151
6	Alzheimer Disease.mp. or Alzheimer Disease/	124,070
7	culinary.mp.	2,604
8	Cooking/ or cook*.ti,ab.	44,173
9	Meals/ or meal.ti,ab.	63,156
10	Food/ or food.ti,ab.	548,362
11	nutrition.mp.	276,241
12	program.ti,ab.	523,303
13	Education/ or education.ti,ab.	570,320
14	Diet Therapy/ or diet* therap*.ti,ab.	15,046
15	intervention.ti,ab.	774,474
16	train*.ti,ab.	701,707
17	Rehabilitation/ or rehabilitation.ti,ab.	199,334
18	1 or 2 or 3 or 4 or 5 or 6	806,976
19	7 or 8 or 9 or 10 or 11	848,896
20	12 or 13 or 14 or 15 or 16 or 17	2,334,373
21	18 and 19 and 20	2,116

No.	Search Terms (CINAHL)	Results (n = 43)
1	(MH “Nervous System Diseases+”) OR (MH “Neurodegenerative Diseases+”) OR (MH “Autoimmune Diseases of the Nervous System+”) OR (MH “Autonomix Nervous System Diseases+”) OR neurological conditions	859,622
2	(MH “Stroke+”) OR stroke	144,282
3	(MH “Brain Injuries+”) OR brain injur*	44,880
4	(MH “Multiple Sclerosis”) OR multiple sclerosis	26,744
5	(MH “Parkinson Disease+”) OR parkinson* disease OR (MH “Parkinsonian Disorders+”)	34,733
6	(MH “Alzheimer’s Disease+”) or alzheimer* disease OR dementia	108,442
7	culinary	4,838
8	(MH “Cooking+”) OR cook*	498
9	(MH “Meals+”) OR meal*	40

10	(MH “Food+”) OR food	317
11	(MH “Nutrition+”) OR (MH “Nutrition Services+”) OR nutrition OR (MH “diet+”) OR diet* OR “diet* change” OR “behavio?r* change”	2
12	program	584,926
13	(MH “Education”) or education	1,593
14	(MH “Diet Therapy+” OR diet* therap* OR nutrition therap*	21
15	intervention OR “skill* buil*” OR (MH “motor skills+”) OR (MH”skill acquisition+”)	573,584
16	train* OR (MH “Cognitive Therapy+”) OR (MH “Cognitive Rehabilitation+”)	342,541
17	(MH “Rehabilitation+”) or rehabilitation	530
18	(MH “Health Education+”) OR health education	196,251
19	(MH “Patient Education+”) OR patient education	104,691
20	(MH “Health Promotion+”) OR health promotion	93,346
21	S1 OR S2 OR S3 OR S4 OR S5 OR S6	943,111
22	S7 OR S8 OR S9 OR S10 OR S11	5,144
23	S12 OR S13 OR S14 OR S15 OR S16 OR S17 OR S18 OR S19 OR S20	1,423,168
24	S21 AND S22 AND S23	43

**No.**	**Search Terms (Embase)**	**Results (n = 5,546)**
1	neurological disease/ or neurological condition.mp.	177,827
2	stroke.mp. or cerebrovascular accident/	644,168
3	brain injury/ or brain injur*.mp. or traumatic brain injury/	196,183
4	multiple sclerosis.mp. or multiple sclerosis/	178,934
5	Parkinson disease/ or Parkinson* disease.mp.	223,538
6	Alzheimer disease/ or alzheimer* disease.mp.	293,932
7	culinary.mp.	3,416
8	cook*.ti,ab. or cooking/	54,455
9	Meals/ or meal.ti,ab.	125,731
10	Food/ or food.ti,ab.	700,954
11	nutrition/ or nutrition.mp.	329,723
12	health program/ or education program/ or program.ti,ab. or nutrition services/	869,594
13	Education/ or education.ti,ab.	1,016,332
14	Diet Therapy/ or diet* therap*.ti,ab.	71,090
15	intervention.ti,ab.	1,170,268
16	train*.ti,ab.	974,082
17	Rehabilitation/ or rehabilitation.ti,ab.	320,302

18	1 or 2 or 3 or 4 or 5 or 6	1,570,292
19	7 or 8 or 9 or 10 or 11	1,090,158
20	12 or 13 or 14 or 15 or 16 or 17	3,625,294
21	18 and 19 and 20	5,546

**Search Terms (Scopus)**	**Results (n = 3,745)**
TITLE-ABS-KEY ("Neurological Condition" OR "Nervous System Disease" OR stroke OR "Brain Injur*" OR "Multiple Sclerosis" OR "Parkinson* Disease" OR "Alzheimer* Disease") AND TITLE-ABS (culinary OR cook* OR meal* OR food OR nutrition) AND TITLE-ABS (program OR education OR "Diet* Therap*" OR intervention OR train* OR rehabilitation) AND (LIMIT-TO (LANGUAGE , "English"))	3,745

**Search Terms (Proquest)**	**Results (n = 1,213)**
**Exact("alzheimer disease" OR "brain injury" OR "brain injuries" OR "multiple sclerosis" OR "parkinson disease" OR "stroke" OR "neurological conditions") AND ((Culinary OR Cook* OR Exact("meals") OR Food OR Nutrition) AND (Program OR Education OR Intervention OR Rehabilitation OR Exact("Nutrition Education" OR "Diet Therapy")))**<br><br>Limit Source Type (Scholarly Journal), Document Type (Evidence Based Healthcare), Language (English)	1,213
